# Reliability of a Trapezium Miniplate with Endoscope-Assisted Internal Fixation in Mandibular Subcondylar Fractures: A Three-Dimensional Analysis

**DOI:** 10.3390/jcm11010207

**Published:** 2021-12-31

**Authors:** Seungwook Jung, Ok Hyung Nam, Yi-Qin Fang, Shavkat Dusmukhamedov, Chunui Lee

**Affiliations:** 1 Department of Oral and Maxillofacial Surgery, Wonju College of Medicine, Yonsei University, Wonju 26426, Korea; Loukas_jung@naver.com (S.J.); qin0302@naver.com (Y.-Q.F.); mr.shavkat595@bk.ru (S.D.); 2 Department of Pediatric Dentistry, School of Dentistry, Kyung Hee University, Seoul 02447, Korea; pedokhyung@gmail.com

**Keywords:** trapezium plate, endoscope, mandibular subcondylar fracture, fracture healing, fracture fixation, open reduction and internal fixation

## Abstract

This study aimed to evaluate the reliability of a trapezium plate for open reduction and internal fixation (ORIF) of mandibular subcondylar fractures with the simultaneous use of an endoscope. We selected and retrospectively studied 18 patients (12 males and 6 females) with unilateral mandibular subcondylar fractures who visited the Wonju Severance Christian Hospital. The mean age of the patients was 43.43 ± 15.76 years. Patients underwent ORIF with trapezium miniplate application through an intraoral incision under general anesthesia. The clinical and radiographic findings of the fractured side were compared with those of the non-operated side at 6 months follow-up. All occlusions became stable, and transient functional disturbances disappeared within 6 months of periodic follow-up. Functional mandibular movement recovered within the normal range, with an average mouth opening of 41.5 mm, protrusion of 7.5 mm, and lateral excursion of 7 mm at 6 months. Radiographic controls and statistical analysis confirmed a decent anatomical reduction in all 18 cases. In conclusion, the use of a trapezium miniplate with endoscope-assisted ORIF in mandibular subcondylar fractures can be useful for fixation and functional recovery.

## 1. Introduction

The optimal treatment protocol for open reduction and internal fixation (ORIF) of mandibular condylar and subcondylar fractures has been debated over decades. Many studies on condylar fracture surgery have introduced various surgical approaches to the condylar region, and verified that the intraoral approach has the least postoperative morbidity [1,2,3]. The application of miniplates fixed with screws is considered the gold standard for ORIF in mandibular surgery [4,5]. Thus, numerous miniplate designs have been introduced during the past decades, and recently, their efficacies have been studied to examine biomechanical stability and surgical simplicity [6,7,8,9]. Previous biomechanical studies have confirmed the reliability of condyle fixation via two miniplates for subcondylar fractures for withstanding the compression and tension forces within physiological limitations. [10,11]. Meyer at al. [12] demonstrated that the trapezoidal plate provided sufficient rigidity for the fixation of subcondylar fractures. Darwich et al. [13] claimed that trapezoidal plates are superior to two miniplates.

Recently, with the emergence of endoscopy-assisted surgery, problems of inadequate surgical view and limited workspace, which are critical disadvantages of intraoral surgery, have been overcome [14,15,16]. Endoscopy-assisted open reduction and internal fixation (EAORIF) of mandibular condylar fractures has resulted in satisfactory postoperative outcomes [17]. A previous study regarding the miniplate system in EAORIF showed that biodegradable plates are as reliable as metal miniplates [18]. However, there are insufficient data for appropriate miniplate designs for EAORIF. Therefore, the present study evaluated the clinical use of a newly developed single trapezium miniplate (Jeil Co., Seoul, Korea) applied through a transoral endoscopy-assisted approach and attempted to present clinical and three dimensional (3D) anaylsis follow-up results.

## 2. Materials and Methods

We retrospectively reviewed patients who had undergone a uniform surgical technique between 2015 and 2018, consisting of an endoscopy-assisted transoral approach with combination fixation using a mono-cortical single trapezium miniplate, with at least 6 months of postoperative follow-up. A total of 18 patients with mandibular subcondylar fractures (12 males and 6 females) between 18–70 years old (mean age 43.83 years old) were included. All patients underwent surgery at the Department of Oral and Maxillofacial Surgery of the Wonju Severance Christian Hospital. The study protocol was reviewed and approved by the Institutional Review Board of Yonsei University Wonju Severance Christian Hospital, Wonju, Korea (CR319018).

**Figure 1:**
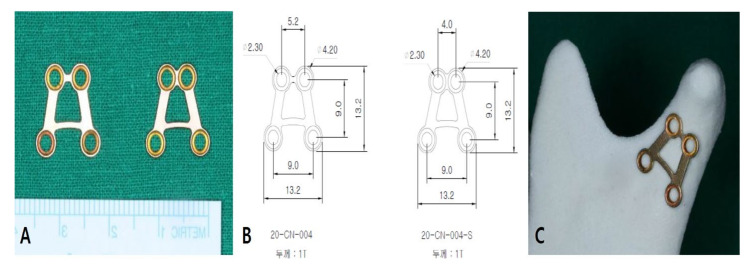
Trapezium plate shape and dimensions. (**A**) Two subtypes of trapezium plate. (**B**) Numerical description of each trapezium plate. (**C**) Trapezium plate applied to a rapid prototype model.

In the trapezium plate, the plate arms were designed according to the ideal line of osteosynthesis introduced in Meyer’s study [19]. Both vertical arms were designed parallel to the compression lines, while the upper and lower horizontal lines were designed along the traction lines. Two types of trapezium plates were designed, which differed in the upper arm size. The arms were similar, but were selectively used in high and low condylar fractures. The plate was 1 mm thick, 13.2 mm long, and 4–5.2 mm wide at the top and 13.2 mm wide at the base. Four holes were located at each corner of the trapezium plate (Figure 1).

**Figure 2:**
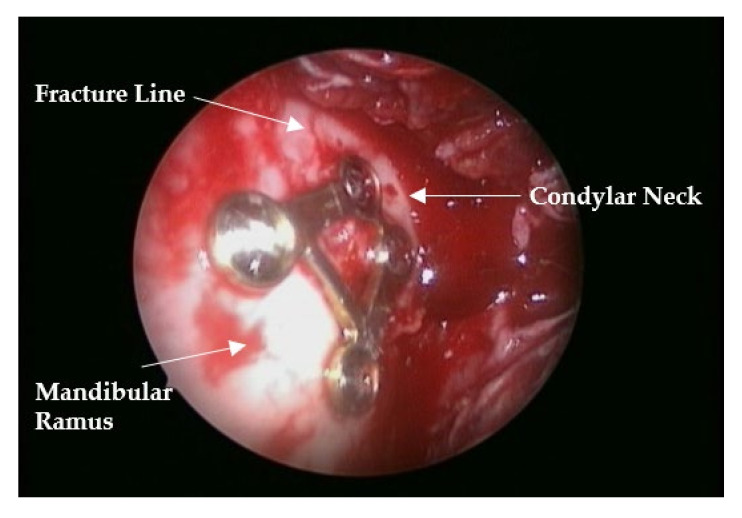
Endoscopic view of the trapezium plate after condylar fracture fixation.

**Figure 3:**
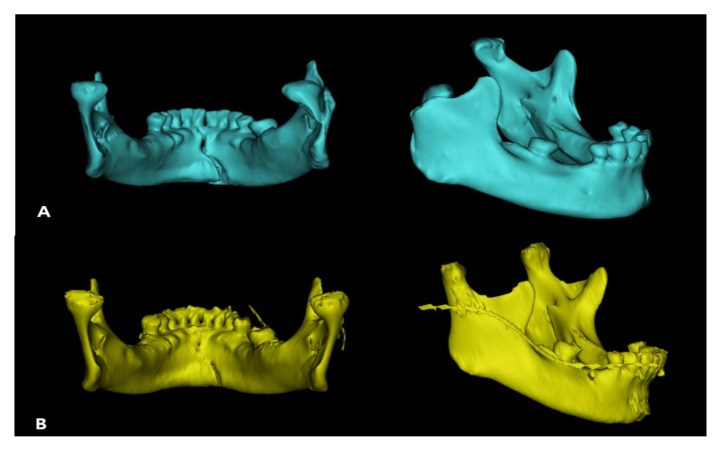
3D reconstruction view. (**A**) Pre-operative 3D view. (**B**) Post-operative 3D view. Using Mimics, an image of the condylar head collapsing inward before surgery was reproduced, and it was confirmed that the condylar head was repositioned after surgery.

All of the patients received general anesthesia. Lidocaine with 1:100,000 epinephrine was injected into the muco-vestibular area along the mandibular ramus. In most cases, anchor screws were located at the upper and lower interdental alveoli for maxillomandibular fixation (MMF). A linear mucosal incision was made over the buccal vestibule and over the anterior border of the ramus, similar to orthognathic surgery. The portion of the mandibular angle and the lateral aspect of the ramus along the fractured subcondylar portion were then exposed by raising the mucoperiosteal flap and masseter muscles using a surgical curette, periosteal elevator, and ramus stripper. A stab incision was made at the ipsilateral overlying skin, according to the level of the fracture line. The trochar system was inserted transbuccally, providing adequate space between the bone and overlying tissue for endoscopic instruments. Under endoscopic visualization of the fractured site, manipulation of the proximal segment for proper reduction was attempted with a trochar shank. When rough reduction was performed, proper occlusion was manually manipulated. To confirm proper reduction and occlusion, a trapezium plate was placed between the trochar shank and the proximal segment of the bone. After temporary intermaxillary fixation via wire engagement on the corresponding upper and lower anchor screws, two holes in the proximal segment were drilled, and screws were inserted into the upper corners of each hole. Thorough determination of the correct anatomical reduction, especially proper alignment of the posterior border of the ramus, was achieved endoscopically. Finally, two holes in the distal fragment were drilled, and screws were inserted. The MMF was removed, and occlusion was confirmed. The outcome of fracture reduction and fixation was verified endoscopically (Karl Storz, Germany) (Figure 2). If necessary, the fixation position was adjusted by slightly loosening and retightening the screws (Figure 3). Wound closure was usually performed using 4-0 vicryl, and a silastic drain was inserted to prevent postoperative hematoma and minor hemorrhage. A liquid diet with intermaxillary elastic engagement was strictly maintained for 2 weeks after the operation. To ensure mouth opening and prevent temporomandibular joint ankylosis, a soft diet was recommended from the third week onwards. The trapezium plate was removed after a minimum of 6 months postoperatively using an intraoral and transcutaneous approach.

**Figure 4:**
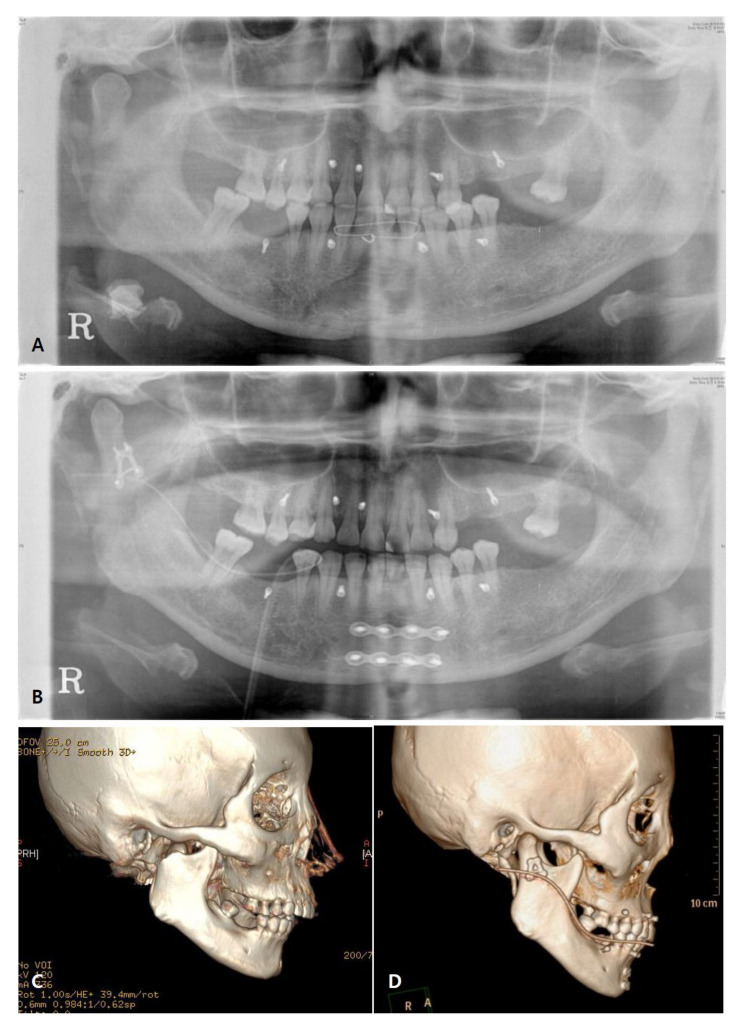
X-ray and 3D facial CT view. (**A**) Pre-operative panoramic view. (**B**) Post-operative panoramic view. (**C**) Pre-operative 3D facial computed tomography scan. (**D**) Post-operative 3D facial computed tomography scan.

**Figure 5:**
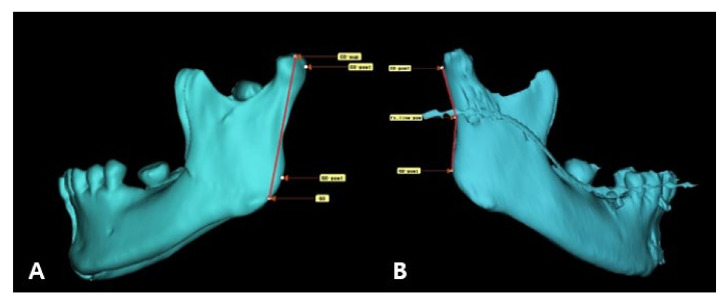
Ramus height and angle of dislocation measured on 3D simulation. (**A**) Pre-operative mandible. (**B**) Post-operative mandible. CO-sup (condylion superius): The most superior point of the condylar head. CO-post (condylion posterius): The most posterior point of the condylar head. GO-post (gonion posterius): The most posterior point on the mandibular angle.

**Figure 6:**
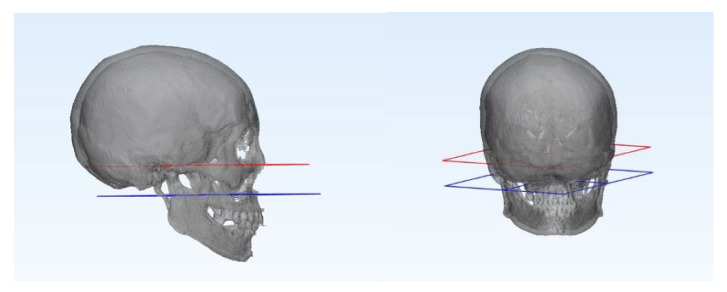
The red plane indicates the FH-plane, orbitale to porion. The blue plane is parallel to the FH-plane and passes through the posterior point of the subcondylar fracture line.

Each patient was followed-up with clinical and radiographic evaluations for at least 6 months. Clinical evaluation included stable occlusion, and trismus evaluation by measurements of the maximum interincisal distance, protrusion, lateral excursion, and mandibular deviation on the mouth opening. Preoperative and postoperative radiographic evaluations were performed using 3D facial mandible computed tomography (CT) in all patients, along with panoramic radiography (Figure 4). Adequate anatomical reduction was evaluated according to Undt et al. [3]. Using 3D simulation in the Mimics medical program, the ramus height was measured from the top point of the condyle to the gonion, and was compared with the non-fractured side. Similarly, the program was used to evaluate the angle of dislocation of the proximal fragment, compared to the contralateral side, using 3D CT DCM files. The angle was measured from the most posterior point of the condylar head to that of the fracture line and that of the mandibular angle (Figure 5). For comparison with the non-fractured side, the FH-plane was used, and the angle was measured by connecting the condylion superius to the point where they met the non-operated proximal condyle by setting a line parallel to the FH-plane and the gonion posterius (Figure 6).

Statistical analysis was performed using the collected data. Descriptive statistics of the patients’ average mouth opening and protrusive and lateral excursion of the mandible were analyzed to evaluate the functional recovery of mandibular movement. The Wilcoxon signed-rank test was performed to compare variables between the fractured and non-fractured sites. The ramus height and angle of dislocation of the operated side and those of the non-fractured side were determined as variables.

## 3. Results

### 3.1. Clinical Evaluation

The operation time ranged between 104 and 128 min, depending on the degree of displacement or the existence of other fracture sites. The plate was easily manipulated during the operation, and the fractured segments were adequately reduced. None of the patients showed chronic inflammation, infection, malunion, or nonunion. The plates were removed in all 18 patients, and no loose screws were found during removal; many of the patients had bone accumulation over the trapezium plate.

### 3.2. Functional Evaluation

During the follow-up period (mean follow-up, 12.2 months; range, 6–24 months), no patient complained of pain or occlusal discomfort. However, postoperatively, two patients complained of occlusal discomfort, which disappeared after intermaxillary elastic engagement 3–5 days after admission. The mean maximum mouth opening was 24.82 ± 1.99 mm immediately after the operation, 32.2 ± 1.32 mm 1 month after the operation, and 41.54 ± 2.21 mm 6 months after the operation.

Protrusive movement and lateral excursion of the mandible varied from 4.5 to 10 mm at the 6-month follow-up timepoint. Lateral deviation on the mouth opening to the fractured side was observed in 11 of the 18 patients. The values of postoperative mandibular movements (maximum mouth opening, protrusion, and lateral excursion) were almost similar to those of the physiological mandibular movement recommended by Okeson [20]. The findings are summarized in Table 1.

### 3.3. Radiographic Evaluation

Anatomical reduction was confirmed via 3D facial mandible computed tomography. The postoperative panoramic view revealed no significant numerical gap between the ramus heights. At 6 months follow-up, the ramus height was retained in most patients (83%). Based on the angle of dislocation in the panoramic view, good anatomic reduction (angle 0° to 20°) was achieved postoperatively in all patients. Radiography revealed no plate fracture or plate bending in any patients.

### 3.4. Statistical Evaluation

The Wilcoxon signed rank test was performed between two subgroups (operated and non-operated sides) in two categories (ramus height and angle of dislocation). At a significance level of 0.05, negative statistical significance was found between the operated and contralateral sides in both categories (Table 2).

## 4. Discussion

With the development of surgical techniques and instruments, miniplates are a dedicated solution for the fixation of mandibular condylar neck and base fractures. Nevertheless, endoscopy-assisted ORIF (EAORIF) should not be indicated for all condylar fractures, as it has limitations in cases with high condylar neck fractures or for comminuted fractures with insufficient bone mass to tolerate screws and plating; in these cases, closed reduction is preferred. However, for cases with proper indication, ORIF of the condylar and subcondylar fractures, rather than closed treatment, should be performed [21,22]. An intraoral approach can decrease the risk of nonesthetic surgical outcomes and postoperative complications such as Frey’s syndrome and facial nerve palsy [3,23,24]. Transoral subcondylar surgery has overcome the limited surgical view via endoscopic assistance, and the need for a small, manageable osteosynthesis device that can replace conventional two-plate bio-stability is increasing [25,26,27]. Trapezium plates for subcondylar fractures have many advantages. First, its shape designs with four arms can be placed at the compression and traction lines. Second, they are easy to manipulate and require less time for placing, but are as stable as the two single miniplate system biomechanics, which requires the drilling of eight holes and screw insertion [8,28,29]. Radiographic findings confirmed proper anatomical reduction, and no permanent functional complications occurred in our patient cohort. Minor deviations were found in five patients, but no functional or esthetic dissatisfaction was noted. Lata et al. [30] compared delta and trapezoidal plates, and found that patients treated with delta plates showed a significant improvement at all intervals in contrast with the trapezoidal plate, which did not show a significant improvement in the mouth opening from 6 weeks to 3 months. Burkhard et al. [9] found that deltoid and trapezoid plates seem to perform equally in the treatment of condyle neck and base fractures. Hochban et al. [4] emphasized that deviations from the anatomically correct position did not affect the function of the temporomandibular joint. In our patient cohort, trismus relief and restoration of the mouth opening were observed within 6 months after operation. Six months after surgery, the amount of MMO, protrusion, and lateral movement showed good results. There is a time difference between the lateral and medial overrides, but it is sufficiently overcome. In our patient cohort, the presence of additional distant fractures of the mandible showed good results. The functional parameters of protrusive and lateral excursion of the mandible were similar between our patients and the various treatment cohorts reported previously [1,31,32]. Kang et al. [33] evaluated the complications of EAORIF of mandibular condylar fractures. The long-term complication rate was much lower than the temporary complication rate, except for cases where malreduction or refracture occurred. EAORIF should be considered reliable for the treatment of condylar fractures, but intensive training and equipment are required.

The statistical insignificance observed between the operated and non-operated sides regarding the ramus height and angle of dislocation indicated consistent operation outcomes, accompanied with anatomic reduction and rigid fixation.

Plate fracture or bending and screw loosening have been observed in previous studies; however, none of these complications occurred in our study. Occasionally, excessive bone accumulation over the trapezium plate interfered with the process of plate and screw removal, which implies vigorous osteogenesis around the fixation site. The two types of trapezium plates differ in the upper horizontal arm length. Therefore, they can be applied to most open subcondylar fractures with various fracture line locations.

The biostability and durability of the trapezium plates were evaluated by Darwich et al. [13]. Five plating techniques (single straight plate, two parallel straight plates, two divergent straight plates, trapezoid plate, and square plate) were compared for the fixation of unilateral mandibular subcondylar fractures with finite element analysis. Among these, the trapezoid plate showed the least micro-mobility and strain on the underlying bones for fixation [29]. Sikora et al. [7] summarized clinical trials regarding the use of various types of 3D condylar mini-plates (trapezoid, deltoid, rhombus, strut, nine-hole trapezoid, and lambda). There are no convincing data that the number of reoperations depends on the type of 3D mini-plate used and clinical fractures of 3D mini-plates are extremely rare, in contrast to the fairly frequent fractures of straight miniplates. The trapezium plate can be applied to various subcondylar fractures. It is accurately designed according to the ideal tension and compression line of the mandible, and can tolerate various forms of loads, with the highest tensile strain occurring at the anterior and lateral surfaces and the highest compressive strains on the posterior surface. Although there is a learning curve for our protocol, the surgical outcome and time efficiency have been verified throughout the past years. The clinical, functional, and radiologic outcomes are consistent with those from previous studies.

However, there are possible complications of this surgical technique. In severely dislocated and comminuted fractures, if the extraoral approach for the endoscopic-assisted treatment of subcondylar fractures is indicated, then facial nerve damage may occur. Subcondylar fractures with medially displaced fragments are more challenging to reduce within the narrow sight under the endoscopic view. So, if an unskilled surgeon performs the operation, re-fracture due to screw loosening, malocclusion, deviation, trismus, and temporomandibular joint disease may occur.

This study has some limitations. First, the number of patients undergoing this surgery is not enough. Second, in the 3D analysis of the position of the condyle after surgery, there is a limit to the analysis of angles in various directions, such as the mesial and lateral side. Moreover, the results for the follow-up of long-term clinical evaluation and functional evaluation are insufficient.

## 5. Conclusions

In conclusion, the use of a trapezium plate with endoscopy-assisted ORIF can be recommended for subcondylar fractures. However, the use of a trapezium with EAORIF has limitations in tolerating screws and plating in cases of high condylar neck fractures or comminuted fractures with insufficient bone mass. Trapezoid shape designs with four arms can be placed at the compression and traction lines; thus, a sufficient fixing force can be achieved even with a small number of screws, with equal stability to other miniplate systems. Considering these advantages, our method can be recommended for the treatment of patients with subcondylar fractures.

## Figures and Tables

**Table 1:** Descriptive statistics.

Variables		Study Population (*n* = 18)
Sex *n* (%)	Men	12 (66.67)
	Women	6 (33.33)
Age (year)	Mean ± SD	43.83 ± 15.76
	Median (min–max)	47.00 (18.00–70.00)
Post-operative MMO (mm)	Mean ± SD	24.82 ± 1.99
	Median (min–max)	24.90 (21.20–27.90)
Post-operative MMO (1 month) (mm)	Mean ± SD	32.23 ± 1.32
	Median (min–max)	32.50 (30.30–34.30)
Post-operative MMO (6 months) (mm)	Mean ± SD	41.54 ± 2.21
	Median (min–max)	42.70 (37.20–47.80)
Mean protrusion (6 months) (mm)	Mean ± SD	7.39 ± 1.60
	Median (min–max)	8.20 (4.00–10.50)
Mean lateral excursion to the right (6 months) (mm)	Mean ± SD	7.28 ± 1.18
	Median (min–max)	7.45 (4.60–9.50)
Mean lateral excursion to the left (6 months) (mm)	Mean ± SD	7.13 ± 1.51
	Median (min–max)	7.05 (4.50–10.20)

**Table 2:** Comparison between the operated and non-operated sides.

Variables		Non-Operated Side (*n* = 18)	Operated Side (*n* = 18)	*p*-Value *
Length (mm)	Mean ± SD	65.68 ± 6.77	65.02 ± 6.48	0.205
	Median (min–max)	65.33 (52.10–79.40)	66.77 (48.16–74.13)	
Angle (n°)	Mean ± SD	166.31 ± 7.10	164.95 ± 8.26	0.099
	Median (min–max)	168.10 (143.00–175.20)	165.30 (142.50–179.50)	

* *p*-value by Wilcoxon signed-rank test.

## Data Availability

Data are available upon reasonable request.
